# Association Between Loneliness Subtypes and Brain Volume in Older Adults With HIV

**DOI:** 10.1093/geroni/igaf122.162

**Published:** 2025-12-31

**Authors:** Moka Yoo-Jeong, Eran Shorer, Raha Dastgheyb, Gregory Wade, Kylie Alm, Arnold Bakker, Tracey Wilson, Leah Rubin

**Affiliations:** Northeastern University, Boston, Massachusetts, United States; Johns Hopkins University, Baltimore, Maryland, United States; Johns Hopkins School of Medicine, Baltimore, Maryland, United States; Johns Hopkins School of Medicine, Baltimore, Maryland, United States; Johns Hopkins University School of Medicine, Baltimore, Maryland, United States; Johns Hopkins University, Baltimore, Maryland, United States; SUNY Downstate Health Sciences University, Brooklyn, New York, United States; Johns Hopkins University School of Medicine, Baltimore, Maryland, United States

## Abstract

Loneliness is a risk factor for cognitive impairment in older adults, with previous studies noting sex differences in both loneliness and brain volume. However, it remains unclear whether there are sex differences in the types of loneliness (i.e., emotional vs. social loneliness) and their relationship to brain volumes in older adults with HIV. Fifty-eight older people with HIV (PWH) over the age of 50 (overall mean age =62, SD = 6; 59% male; 76% Black) completed the De Jong Gierveld Loneliness Scale and underwent T1-weighted structural magnetic resonance imaging of the brain. Brain volumes from the left and right hemispheres were summed to achieve total brain volumes for total cortical gray matter (GM), frontal, parietal, temporal, occipital, subcortical, and cerebellar GM as well as cerebral and cerebellar white matter (WM). A series of multivariable regression models were conducted to assess the association between loneliness subtypes and brain volumes, controlling for relevant covariates. While loneliness did not differ significantly between sexes, associations between loneliness and brain volumes were only evident in men with HIV. Specifically, greater emotional loneliness was associated with reduced brain volumes in cortical, frontal, parietal, occipital, and subcortical GMs (R’s=-0.418 to -4.80, p’s=0.008-0.024), and in cerebellar WM (R=-0.431, p = 0.020). Social loneliness was not associated with brain volumes in either sex. Findings suggest that emotional loneliness is associated with reduced brain volumes in older men with HIV, highlighting the need for further investigation into sex-specific patterns of loneliness to better preserve cognitive function.

